# Prediction of outcome after diagnosis of metachronous contralateral breast cancer

**DOI:** 10.1186/1471-2407-11-114

**Published:** 2011-03-30

**Authors:** Sara Alkner, Pär-Ola Bendahl, Mårten Fernö, Jonas Manjer, Lisa Rydén

**Affiliations:** 1 Department of Oncology, Clinical Sciences, Lund, Lund University, Sweden; 2 Department of Surgery, Clinical Sciences, Malmö, Lund University, Sweden; 3 Department of Surgery, Clinical Sciences, Lund, Lund University, Sweden; 4 Skåne Department of Oncology, Skåne University Hospital, Sweden; 5 Department of Surgery, Malmö, Skåne University Hospital, Sweden; 6 Department of Surgery, Lund, Skåne University Hospital, Sweden

**Keywords:** metachronous breast cancer, contralateral breast cancer, prognosis, detection, adjuvant therapy

## Abstract

**Background:**

Although 2-20% of breast cancer patients develop a contralateral breast cancer (CBC), prognosis after CBC is still debated. Using a unique patient cohort, we have investigated whether time interval to second breast cancer (BC2) and mode of detection are associated to prognosis.

**Methods:**

Information on patient-, tumour-, treatment-characteristics, and outcome was abstracted from patients' individual charts for all patients diagnosed with metachronous CBC in the Southern Healthcare Region of Sweden from 1977-2007. Distant disease-free survival (DDFS) and risk of distant metastases were primary endpoints.

**Results:**

The cohort included 723 patients with metachronous contralateral breast cancer as primary breast cancer event. Patients with less than three years to BC2 had a significantly impaired DDFS (p = 0.01), and in sub-group analysis, this effect was seen primarily in patients aged <50. By logistic regression analysis, patients diagnosed with BC2 within routine follow-up examinations had a significantly lower risk of developing metastases compared to those who were symptomatic at diagnosis (p < 0.0001). Chemotherapy given after breast BC1 was a negative prognostic factor for DDFS, whereas endocrine treatment and radiotherapy given after BC2 improved DDFS.

**Conclusions:**

In a large cohort of patients with CBC, we found the time interval to BC2 to be a strong prognostic factor for DDFS in young women and mode of detection to be related to risk of distant metastases. Future studies of tumour biology of BC2 in relation to prognostic factors found in the present study can hopefully provide biological explanations to these findings.

## Background

Within their lifetime, 2-20% of breast cancer patients develop a new tumour in their contralateral breast [1-3]. These contralateral breast cancers (CBC) are called synchronous if the second tumour (BC2) develops within a short time interval from the first tumour (BC1), and metachronous if the time interval between tumours is longer. In line with several previous studies, we define metachronous tumours as CBC diagnosed at least three months after BC1 [3-5]. However, a clear cut-off time is not defined in the literature. CBC is today treated as a new primary tumour (two individual tumours), but the biological relationship between BC1 and BC2, and the impact of a second primary tumour on prognosis is debated [4,6-18]. Previous studies indicate that prognosis after CBC could be associated with age, time interval between BC1 and BC2, mode of detection of BC2, and adjuvant treatment for BC1 [4,15-17,19]. However, despite women with a history of breast cancer have a high lifetime risk of developing CBC, the annual risk remains at a relative low level of 0.5-1%. A long follow-up time is hence needed in order to obtain a large cohort of patients with CBC.

For this study data was abstracted from individual charts for all patients diagnosed with metachronous CBC in the Southern Healthcare Region of Sweden (a region with 1.7 million inhabitants) from 1977 to 2007. This gave us a unique cohort, including more than 700 patients from multiple medical centres, providing information on patient and tumour characteristics, treatment, and outcome. The aims of this study were to examine prognosis after CBC in relation to time interval between BC1 and BC2, mode of detection of BC2, and treatment for BC1.

## Methods

### Study Cohort

Inclusion criteria were patients within the Southern Swedish Healthcare Region with two breast cancers reported in the Swedish Cancer Register, with the second tumour diagnosed between 1977 and 2007. The Swedish Cancer Register is a nationwide database including the International Classification of Diseases code and date of diagnosis. The study cohort includes patients from 14 hospitals (Lund, Malmö, Helsingborg, Ängelholm, Landskrona, Ystad, Trelleborg, Hässleholm, Kristianstad, Växjö, Ljungby, Halmstad, Karlshamn, and Karlskrona) within the Southern Healthcare Region of Sweden. All hospitals were active members of the South Sweden Breast Cancer Group, established in 1977, and used the common guidelines for diagnosis, treatment, and follow-up. The follow-up program included annual physical examination and mammogram. From 1977 to 1995 the recommended follow-up period was ten years, which was down-scaled to five years from 1995 to 2002, and three years from 2002 onwards. The regular surveillance program was additionally followed by admittance to the screening program for mammographic examinations every 24 months.

**Figure 1:**
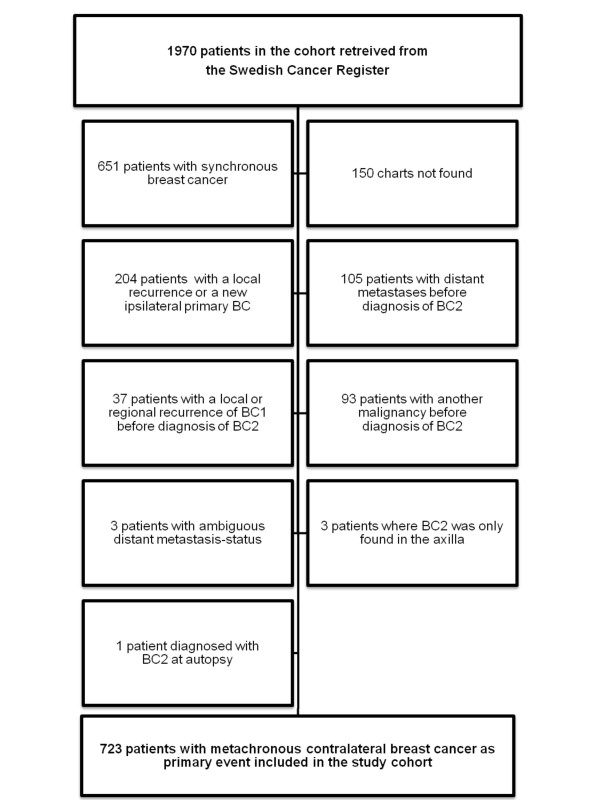
Flow-chart of inclusion *vs. *exclusion in the study cohort

The cohort retrieved from the register initially included 1970 patients. The flow-chart of the study is given in Figure 1. After exclusion according to predefined exclusion criteria, our cohort included 723 patients with metachronous contralateral breast cancer as primary event. For patients with multiple exclusion criteria, the first criterion mentioned in the chart is listed in Figure 1.

### Data abstraction of clinical information

From September 2007 to November 2009, data was abstracted from individual charts (clinical notes, pathology-, and X-ray-records) in a systematised manner, using a predefined protocol. The protocol was designed at the Department of Medical Epidemiology and Biostatistics, KI Stockholm, for collecting data from patients with CBC. Individual charts at the Departments of surgery as well as the Departments of oncology (Lund and Malmö) were retrieved, in order to optimise data abstraction and minimise patients lost to follow-up. The protocol used included data on mode of detection as well as surgical and oncological treatment for BC1 and BC2. Patients diagnosed within a follow-up programme were considered asymptomatic at time of diagnosis, whereas patients who first noted symptoms themselves and thereafter contacted their physicians were considered symptomatic. Patients were considered to have received endocrine treatment only if they had continued treatment for at least three months. Due to the long follow-up period of the study cohort, oestrogen receptor status for both tumours were available for less than half of the patients, and histological grade for less than one third. Additionally, scoring methods to determine histological grade differed during the study follow-up period and between various pathology departments. Neither oestrogen receptor status, nor histological grade, was hence used for further statistical analysis in the present study.

Previous studies use different time intervals to BC2 to separate early from late metachronous CBC. A time interval of three years has been used for a multitude of previous studies [13,15,16], and this interval was hence selected for all analysis presented in this report. Unless otherwise stated, age refers to the age at diagnosis of BC1, in line with earlier studies [4,14,16,20]. To examine the effect of calendar period at diagnosis of CBC on prognosis, we divided the material into three calendar periods, 1977 to 1986, 1987 to 1996, and 1997 to 2007.

### Ethical Considerations

This project has been approved by the ethical committee of Lund University (LU 240-01). All information and data was handled confidentially, and evaluation of information linked to patients was carried out in accordance with the Swedish Personal Data Act (*Personuppgiftslagen *in Swedish).

### End-points and follow-up

Distant disease-free survival (DDFS) was chosen as primary end-point of the study. DDFS includes development of distant metastases (visceral, skeletal, brain, or cutaneous metastases) as a primary event. Loco-regional recurrences were not regarded as events in analysis of DDFS, and event-free survival was measured from diagnosis of CBC. Survival from BC1 was not considered since that would automatically prolong the follow-up until event for patients with a longer time interval between tumours, and hence bias the results. If no prior event was recorded, DDFS was calculated to the last follow-up date in the patient's individual chart. For patients who developed a malignancy other than breast cancer after diagnosis of CBC, the diagnosis date of this malignancy was considered to be the last follow-up date.

### Statistical Analysis

For statistical calculations, the software package Stata 10.1 (StataCorp. 2008. College Station, TX, USA) was used. Kaplan-Meier plots were used to describe DDFS and the log-rank test was used to evaluate hypotheses of equal survival. Kaplan-Meier curves were curtailed when less than five individuals remained at risk. Cox regression was used to estimate hazard ratios (HR), and assumptions of proportional hazards were checked graphically. Retrospective studies of prognosis in relation to mode of detection could be affected by lead-time bias (earlier detection leads to a longer follow-up until event, even if disease progression is the same). To avoid this we looked at the risk of distant metastasis as a primary event instead of DDFS in relation to mode of detection. Logistic regression was therefore used to compare risk of metastasis in different sub-groups. Multivariate analyses presented do not include patients with missing values for any of the variables included. However, the analyses were repeated for all patients by treating the missing category for discrete variables as a separate category and by imputing the sample mean over all patients for continuous variables. To assess whether the effect of time interval to BC2 or mode of detection differed with age, a Cox model was used with a term for interaction between each of these factors and age. Age was categorised as over or equal to *vs. *under age 50 at diagnosis of BC1. All p-values correspond to two-sided tests and values less than 0.05 were considered significant.

## Results

### Clinical information

Patient, tumour and treatment characteristics are described in Table 1. The median duration of follow-up after BC2 was 5.6 years (IQR 2.0-9.1) for all patients without an event. Median duration of follow-up for patients with a time interval to BC2 less than three years was 7.0 years, and for those with a time interval to BC2 of three years or more, 5.4 years. The median time to development of metastasis after BC2 was 2.2 years.

**Table 1:** Patient, tumour, and treatment characteristics in relation to development of metastasis after BC2

	First breast cancer, No (%)		Second breast cancer, No (%)	
	**Metastasis**	**No metastasis**	**Metastasis**	**No metastasis**
**No = 723**	**No = 210**	**No = 513**	**No = 210**	**No = 513**

**Diagnosis**				
<1977	37 (18)	97 (19)	0	0
1977-1986	66 (31)	161 (31)	53 (25)	95 (19)
1987-1996	79 (38)	191 (37)	78 (37)	179 (35)
1997-2007	28 (13)	64 (12)	79 (38)	239 (47)

**Age (years)**				
*Median (range)*	*52 (27-85)*	*60 (30-90)*	*60 (32-92)*	*70 (32-98)*
<50	94 (45)	123 (24)	50 (24)	34 (7)
≥50	116 (55)	390 (76)	160 (76)	479 (93)

**Menopausal status**				
Premenopausal	100 (51)	132 (28)	29 (16)	32 (7)
Postmenopausal	96 (49)	336 (72)	148 (84)	440 (93)
Missing	14	45	33	41

**Node status**				
N0	105 (53)	330 (72)	97 (50)	292 (73)
N+	92 (47)	130 (28)	96 (50)	106 (27)
Missing	13	53	17	115

**Size (mm)**				
*Median (range)*	*20 (1-90)*	*15 (1-100)*	*18 (1-110)*	*14 (1-90)*
Missing	28	50	17	20

**Surgery**				
Modified radical mastectomy	162 (78)	362 (71)	158 (75)	340 (66)
Partial mastectomy	44 (21)	149 (29)	45 (21)	161 (31)
No Surgery	1 (0.5)	0	6 (3)	12 (2)
Missing	3	2	1	0

**Radiotherapy**				
No	56 (27)	213 (42)	122 (58)	360 (70)
Yes	153 (73)	293 (58)	87 (42)	152 (30)
Missing	1	7	1	1

**Chemotherapy**				
No	172 (82)	473 (94)	178 (85)	491 (96)
Yes	37 (18)	32 (6)	31 (15)	21 (4)
Missing	1	8	1	1

**Endocrine treatment **No	158 (76)	391 (77)	132 (64)	296 (58)
Yes	51 (24)	114 (23)	74 (36)	215 (42)
Missing	1	8	4	2

**Abbreviations: No ***number*, **N+ ***lymph node metastases*, **N0 ***no lymph node metastases*, **node status ***lymph node status*.

For patients operated for BC1, 524 patients (73%) received surgery with a modified radical mastectomy and 193 (27%) breast conserving surgery. The corresponding numbers for BC2 were 498 (71%) with modified radical mastectomy and 206 (29%) breast conserving surgery. Endocrine treatment included tamoxifen, aromatase inhibitors, and/or ooforectomy. For over 80% of patients the endocrine treatment given was tamoxifen.

Mode of detection of BC2 was known for 692 patients. Of these, 250 patients first noted the symptoms themselves, 97 patients were diagnosed by clinical examination, 257 patients by clinical mammography during follow-up, and 70 patients by screening mammography after re-admittance to the screening programme. Eighteen patients were diagnosed by other means (such as prophylactic mastectomy, or examination for other symptoms). These patients, along with 31 patients with missing data, were excluded from further analysis in regard to mode of detection. Patients who first noted symptoms themselves were considered symptomatic, while those diagnosed with clinical examination or mammography were considered asymptomatic at diagnosis.

### Time interval between first and second breast cancer in relation to prognosis

The time interval between BC1 and BC2 was 0.30-36 years, with a median of 6.7 years. A time interval of less than five years was most common (42%) between diagnosis of BC1 and BC2, with a decline in the percentage of patients diagnosed with BC2 the longer the time interval to BC2 (5-9 years 24%, 10-14 years 16%, 15-19 years 9%, 20-24 years 5%, 25-29 years 2%, 30-34 years 1%, and ≥35 years 0%). Patient and tumour characteristics in relation to time interval to BC2 are described in Table 2.

**Table 2:** Patient, tumour, and treatment characteristics in relation to time interval to and mode of detection of the second breast cancer

	Time interval to second breast cancer				**Mode of detection of second breast cancer **^a^			
	**First breast cancer, No (%)**		**Second breast cancer, No (%)**		**First breast cancer, No (%)**		**Second breast cancer, No (%)**	

	**<3 years**	≥**3 years**	**<3 years**	≥**3 years**	**Symptomatic**	**Asymptomatic**	**Symptomatic**	**Asymptomatic**
	**No = 200**	**No = 523**	**No = 200**	**No = 523**	**No = 250**	**No = 424**	**No = 250**	**No = 424**

**Diagnosis of BC2**								
<1977	1 (1)	133 (25)	0	0	70 (28)	55 (13)	0	0
1977-1986	59 (30)	168 (32)	48 (24)	100 (19)	77 (31)	132 (31)	57 (23)	79 (19)
1987-1996	84 (42)	186 (36)	80(40)	177 (34)	84 (34)	174 (41)	89 (36)	150 (35)
1997-2007	56 (28)	36 (7)	72 (36)	246 (47)	19 (8)	63 (15)	104 (42)	195 (46)

**Age**								
*Median (range)*	*59 (39-89)*	*57 (27-90)*	*60 (32-91)*	*69 (36-98)*	*54 (27-89)*	*59 (30-87)*	*66 (35-97)*	*68 (32-93)*
<50 years	55 (28)	162 (31)	48 (24)	36 (7)	96 (38)	110 (26)	45 (18)	34 (8)
≥50 years	145 (73)	361 (69)	152 (76)	487 (93)	154 (62)	314 (74)	205 (82)	390 (92)

**Node status**								
N0	111 (60)	324 (69)	108 (65)	281 (66)	161 (72)	253 (65)	117 (56)	251 (71)
N+	75 (40)	147 (31)	59 (35)	143 (34)	62 (28)	138 (35)	91 (44)	104 (29)
Missing	14	52	33	99	27	33	42	69

**Size (mm)**								
*Median (range)*	*17 (1-100)*	*17 (1-70)*	*14 (1-110)*	*15 (1-85)*	*18 (1-90)*	*17 (1-100)*	*19 (1-110)*	*13 (1-90)*
Missing	6	72	10	27	38	32	12	19

**Radiotherapy**								
No	89 (45)	180 (35)	121 (61)	361 (69)	96 (39)	153 (36)	158 (63)	283 (67)
Yes	111 (56)	335 (65)	79 (40)	160 (31)	152 (61)	268 (64)	91 (37)	141 (33)
Missing	0	8	0	2	2	3	1	0

**Chemotherapy**								
No	167 (84)	478 (93)	177 (89)	492 (94)	227 (92)	376 (89)	220 (88)	402 (95)
Yes	33 (17)	36 (7)	23 (12)	29 (6)	20 (8)	45 (11)	29 (12)	22 (5)
Missing	0	9	0	2	3	3	1	0

**Endocrine treatment**								
No	148 (74)	401 (78)	124 (63)	304 (59)	202 (82)	313 (74)	146 (59)	249 (59)
Yes	52 (26)	113 (22)	74 (37)	215 (41)	45 (18)	108 (26)	102 (41)	172 (41)
Missing	0	9	2	4	3	3	2	3

**Mode of detection of BC2**								
Symptomatic			57 (31)	193 (40)				
Asymptomatic			129 (69)	295 (60)				
Missing			14	35				

**Time interval to BC2**								
*Median (range)*							*9.1 (0.37-36)*	*5.8 (0.30-34)*
<3 years							57 (23)	129 (30)
≥3 years							193 (77)	295 (70)

^a ^49 patients were excluded from analyses regarding mode of detection due to missing data or diagnosis by other means than mammography, clinical examination or symptoms noted by the patient.

**Abbreviations: N+ ***lymph node metastases*, **N0 ***no lymph node metastases*, **No ***number*, **node status ***lymph node status.*

When exploring the time interval to BC2 as a continuous variable, we found a significantly improved DDFS per year the longer the time interval to BC2 (HR = 0.97, p = 0.002, 95% CI 0.94-0.99). This result remained statistically significant in multivariate analysis adjusted for age, calendar period, mode of detection of BC2, tumour size, lymph node status, and treatment for BC1 and BC2 (HR = 0.94, p < 0.001, 95% CI 0.91-0.97). When dividing the time interval ≥3 years to BC2 into two separate categories, 3-9 years and ≥10 years, we found that DDFS seems to improve continuously with increasing time interval to BC2. Compared to patients with a time interval to BC2 of ≥10 years (the selected reference group), patients with a time interval of 3-10 years had a HR of 1.3 (p = 0.1, 95% CI 0.95-1.9), whereas patients with a time interval of less than three years had a HR of 1.7 (p = 0.003, 95% CI 1.2-2.4).

**Figure 2:**
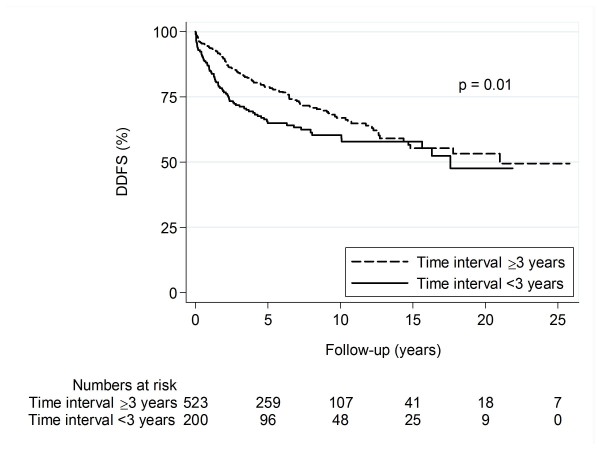
**Distant disease-free survival in relation to time interval to BC2**. **Abbreviations**: **BC2 ***the second breast cancer*, **DDFS ***distant disease-free survival*, **Interval ***time interval to BC2.*

Patients with a time interval to BC2 of less than three years had a significantly impaired DDFS compared to patients with a time interval of more than three years (Figure 2, Table 3). Statistical significance remained in multivariate analysis adjusted for age, calendar period, mode of detection of BC2, tumour size, lymph node status, and treatment for BC1 and BC2 (Table 3).

**Table 3:** Cox-regression analysis for distant metastasis in relation to time interval to the second breast cancer

Age at BC1	Time interval to	Cases	Metastasis	Metastasis/100.000	HR	HR*	HR**
	BC2	No	No (%)	person-years	(95% CI)	(95% CI)	(95% CI)
**All**	<3 years	200	74 (37)	5800	1.4 (1.1-1.9)	1.6 (1.1-2.2)	1.6 (1.1-2.3)
**No = 723**	≥3 years	523	136 (26)	4100	1.0	1.0	1.0
					p = 0.01	p = 0.009	p = 0.01

**< 50 years**	<3 years	55	34 (62)	11000	2.2 (1.4-3.4)	2.0 (1.2-3.4)	2.2 (1.2-3.8)
**No = 217**	≥3 years	162	60 (37)	4900	1.0	1.0	1.0
					p < 0.0001	p = 0.01	p = 0.006

≥ **50 years**	<3 years	145	40 (28)	4100	1.2 (0.78-1.7)	1.5 (0.92-2.3)	1.3 (0.77-2.2)
**No = 506**	≥3 years	361	76 (21)	3700	1.0	1.0	1.0
					p = 0.5	p = 0.1	p = 0.3

* Adjusted for calendar period (1977-1986, 1987-1996, 1997-2007), age (under *vs. *over 50 years at diagnosis of BC1), mode of detection of BC2 (symptomatic *vs. *asymptomatic), size (mm) of BC1 and BC2, and node status (N0 *vs. *N+) for BC1 and BC2. Multivariate analyses in subgroups divided by age are not adjusted for age again. No = 471, 158 patients <50 years and 313 ≥50 years.

** Adjusted for factors listed under * and in addition: treatment for BC1 and BC2 (radiotherapy, chemotherapy, endocrine treatment, yes *vs. *no for each of the variables). Multivariate analyses in subgroups divided by age are not adjusted for age again. No = 468, 157 patients <50 years and 311 ≥50 years.

**Abbreviations**: **BC1 ***first breast cancer*, **BC2 ***second breast cancer***, CI ***confidence interval*, **HR ***hazard ratio*, **N+ ***lymph node metastases*, **N0 ***no lymph node metastases*, **No ***number*, **node status ***lymph node status.*

**Figure 3:**
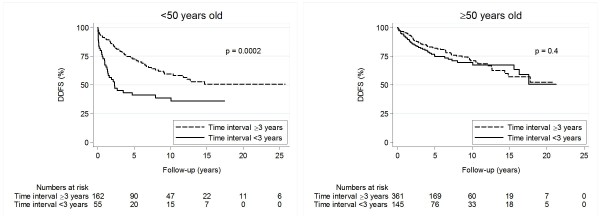
**Distant disease-free survival in relation to age and time interval to BC2**. Age refers to age at diagnosis of BC1. **Abbreviations**: **BC2 ***the second breast cancer*, **DDFS ***distant disease-free survival*, **Interval ***time interval to BC2.*

Subgroup analysis showed that women younger than 50 years when diagnosed with BC1 had a significantly worse DDFS if the time interval to BC2 was less than three years (Figure 3, Table 3). This result remained statistically significant in multivariate analysis (Table 3). However, for women of 50 years or older, no significant difference was seen in regard to time interval to BC2 - neither in univariate- nor multivariate analysis (Figure 3, Table 3). When using a Cox model with main effects for age and time interval to BC2, and a term for the interaction between these variables, the interaction was statistically significant (HR = 0.53, p = 0.03, 95% CI 0.30-0.94). However, when the data was adjusted for calendar period, mode of detection of BC2, tumour size, lymph node status, and treatment for BC1 and BC2, no statistical significance remained. Multivariate analyses were repeated to include patients with missing values, indicating similar results as above.

Menopausal status at diagnosis of both BC1 and BC2 was known for 611 patients (85%). Repeating analyses according to menopausal status, results for premenopausal patients and patients who switched menopausal status between tumours were similar to those observed for patients <50 years at BC1, while results for postmenopausal patients were similar to those observed for patients ≥50 years (data not shown). Out of the 198 patients who were <50 years old at BC1, only 9 (5%) were postmenopausal, whereas for the 466 patients ≥50 years of age at BC1, only 43 (9%) were premenopausal. An age-limit of 50 years hence seems to effectively separate pre- and postmenopausal women.

### Mode of detection in relation to prognosis

For 424 (63%) of the patients, CBC was diagnosed within a follow-up programme, whereas 250 patients (37%) first noted symptoms themselves and thereafter contacted their physician. The median duration of follow-up after BC2 for patients without an event was 5.4 years for symptomatic patients, and 5.7 years for asymptomatic patients. Patient and tumour characteristics are described in Table 2.

Patients with symptoms at diagnosis were younger and had a longer time interval between tumours. Additionally their contralateral tumours were larger, and more often combined with the occurrence of lymph node metastases (Table 2). Using logistic regression, a significantly higher risk of developing metastases was seen in patients who were symptomatic at diagnosis (Table 4). The statistical significance remained when adjusting for age, calendar period, tumour size, node status, and treatment for both tumours. This suggests that finding BC2 at an earlier stage is not the only factor improving prognosis in patients asymptomatic at diagnosis. Multivariate analyses were repeated to include patients with missing values. All analyses yielded similar results as described above.

**Table 4:** Logistic regression analysis for risk of metastasis in relation to mode of detection and time interval to the second breast cancer

Patients groups	Mode of detection	Cases	Metastasis	OR	OR*	OR**
		No	No (%)	(95% CI)	(95% CI)	(95% CI)
**All**	Symptomatic	250	98 (39)	2.1 (1.5-3.0)	2.0 (1.3-3.1)	2.1 (1.3-3.3)
**No = 674**	Asymptomatic	424	99 (23)	1.0	1.0	1.0
				p < 0.0001	p = 0.002	p = 0.002

**< 3 years to BC2**	Symptomatic	57	30 (53)	2.4 (1.3-4.5)	1.7 (0.71-4.1)	1.7 (0.67-4.2)
**No = 186**	Asymptomatic	129	41 (32)	1.0	1.0	1.0
				p = 0.008	p = 0.2	p = 0.3

≥ **3 years to BC2**	Symptomatic	193	68 (35)	2.2 (1.5-3.4)	2.2 (1.3-3.8)	2.3 (1.3-4.0)
**No = 488**	Asymptomatic	295	58 (20)	1.0	1.0	1.0
				p < 0.0001	p = 0.003	p = 0.003

**< 50 years at BC1**	Symptomatic	96	53 (55)	2.2 (1.3-3.9)	1.5 (0.68-3.3)	1.4 (0.62-3.4)
**No = 206**	Asymptomatic	110	39 (35)	1.0	1.0	1.0
				p = 0.005	p = 0.3	p = 0.4

≥**50 years at BC1**	Symptomatic	154	45 (29)	1.7 (1.1-2.7)	2.2 (1.2-3.9)	2.3 (1.3-4.1)
**No = 468**	Asymptomatic	314	60 (19)	1.0	1.0	1.0
				p = 0.01	p = 0.006	p = 0.006

* Adjusted for calendar period (1977-1986, 1987-1996, 1997-2007), age (under *vs. *over 50 years at diagnosis of BC1), size (mm) of BC1 and BC2, and node status (N0 *vs. *N+) for BC1 and BC2. No = 471, 138 patients with <3 years to BC2, and 333 patients with ≥3 years to BC2, 158 patients <50 years and 313 ≥50 years.

** Adjusted for HR* and in addition: treatment for BC1 and BC2 (radiotherapy, chemotherapy, endocrine treatment, yes *vs. *no for each of the variables.). No = 468, 137 patients with <3 years and 331 patients with ≥3 years to BC2, 157 patients <50 years and 311 ≥50 years.

**Abbreviations**: **BC1 ***first breast cancer*, **BC2 ***second breast cancer***, CI ***confidence interval*, **DDFS ***distant disease-free survival*, **N+ ***lymph node metastases*, **N0 ***no lymph node metastases*, **No ***number*, **node status ***lymph node status*, **OR ***odds ratio*

Mode of detection remained a significant risk factor for development of distant metastases when the time interval ≥3 years to BC2 was divided into 2 separate categories; 3-9 years and ≥10 years, with a time interval of less than three years selected as reference group (3-9 years; HR = 2.2, p = 0.005 95% CI 1.3-4.0. ≥10 years; HR = 3.0, p = 0.001, 95% CI 1.5-5.8).

When comparing mode of detection for patients diagnosed by clinical examination with those diagnosed by mammography, patients diagnosed by mammography were younger, had smaller tumours, and more seldom lymph node metastases of BC2 (data not shown). Additionally the risk of later metastasis was slightly, though not significantly, higher for patients diagnosed by clinical examination

### Association between adjuvant treatment and prognosis

A multivariate Cox-regression analysis adjusted for time interval between tumours, calendar period of diagnosis, mode of detection of BC2, age at BC1, lymph node status, tumour size, and treatment for BC1 and BC2, showed chemotherapy given for BC1 to be an independent negative prognostic factor for DDFS (HR = 2.1, p = 0.008, 95% CI 1.2-3.6). Radiotherapy delivered after BC1 did not reach statistical significance as a prognostic factor for DDFS (HR = 1.5, p = 0.07, 95% CI 0.98-2.3), and endocrine adjuvant treatment given after BC1 was not linked to DDFS (HR = 1.2, p = 0.5, 95% CI 0.74-1.9). However, adjuvant radiotherapy (HR = 0.6, p = 0.007, 95% CI 0.41-0.87) and endocrine treatment (HR = 0.5, p = 0.001, 95% CI 0.33-0.74) after BC2 were positive prognostic factors, whereas chemotherapy given after BC2 had no effect on DDFS (HR = 0.88, p = 0.6, 95% CI 0.50-1.5). Multivariate analyses were repeated to include patients with missing values with similar results as described above.

## Discussion

Using a large population-based cohort, including patient, tumour, and treatment information, we have studied prognosis after CBC in relation to time interval to, and mode of detection of BC2. A short time interval to BC2 was proven to be a significant negative prognostic factor, supporting earlier studies [4,15-17]. However sub-group analysis indicates that this effect is seen only for younger women. Data additionally indicate that women diagnosed by routine follow-up examination, have a significantly lower risk of developing metastases at a later stage.

In line with our data, previous studies have found a short time interval to BC2 to be associated with an impaired prognosis [4,15-17]. This study includes extended individual data from a large population-based cohort and strongly validate that a short time interval to BC2 is a negative prognostic factor. Time interval to BC2 was analysed both as a continuous and as a dichotomised variable with a cut-point of three years, and yielded independent prognostic information by both methods. Furthermore, previous results have differed when comparing prognosis after synchronous, metachronous, and unilateral breast cancer [10]. However, there have been indications that synchronous breast cancer (defined as CBC diagnosed within 3-12 months after BC1) might result in a worsened prognosis compared to metachronous breast cancer [10,11,18].

The underlying cause for the impaired prognosis observed for CBC diagnosed within a short time interval from BC1 is unclear. One potential explanation could be that these tumours more often represent a metastatic spread of BC1. Comparisons of genetic alternations in bilateral breast cancer have shown that although most CBCs represent a new primary tumour, contralateral spreading from BC1 does occur [6-9]. A short time interval to BC2 has also been found to correlate with increased genetic and morphological similarities between bilateral tumours [21,22]. This could suggest a higher prevalence of metastatic spread, but could also be a result of these tumours having developed in a similar biological environment. Imyanitov et al. found the highest correlation between tumours in women who developed both tumours while premenopausal [21]. If this correlation results from a higher percentage of contralateral metastatic spread in these patients, it could partially explain the different effects of time interval to BC2 observed for the different age categories in our study, where most patients (95%) diagnosed when under 50 years of age were premenopausal. However, Imyanitov et al. observed the lowest correlation between bilateral tumours separated by menopause, making hormonal environment at the time of development another likely cause to similarities *vs. *differences observed [21].

Another potential explanation could be that CBC diagnosed during, or soon after, adjuvant treatment has developed resistance to treatment and a more aggressive phenotype. The incidence of CBC in Sweden has decreased since the early 1980s [4]. This observation is potentially resulting from an increased use of adjuvant treatment, reducing the risk of CBC [2,3,23-25]. However, during the same time period mortality increased for those women who did develop CBC [4], which could reflect a treatment-escape phenomenon once therapy has failed to prevent a second tumour. CBC developed after tamoxifen treatment is also ER-negative to a larger extent [2,26-28], indicating that treatment given for BC1 affect the biology of BC2. If younger patients have received more adjuvant treatment, this might hence be one explanation to why the time interval between tumours is of greater importance in younger patients. In the present cohort we found support for this theory in that BC1 in younger patients was more often treated with radiotherapy and chemotherapy, while older women received more endocrine therapy (data not shown).

In this study chemotherapy given for BC1 was an independent negative prognostic factor, though this was not seen if chemotherapy had been given for BC2. Adjuvant endocrine treatment after BC1, however, had no effect on prognosis after BC2. Radiotherapy and endocrine treatment were positive prognostic factors if given after BC2, but not if given after BC1. Similar results have been seen in a study by Hartman et al and this could be interpreted as an indication that a CBC diagnosed after prior chemotherapy is more aggressive [4]. However, choice of adjuvant therapy is strongly dependent on tumour stage and biology, and patients selected to receive chemotherapy are those with the worst predicted prognosis. The survival analysis is adjusted for several prognostic factors including tumour stage and all treatment given. There are however still other factors, affecting prognosis and choice of therapy (hormone receptor status, histological grade, etc.) which are not provided for all patients in the present study, including patients for several decades. Hence, although these results are interesting and in line with those found by Hartman et al [4], they need to be interpreted with caution and further studies are required. The effect on prognosis by adjuvant endocrine treatment for BC1 is not fully elucidated since the cohort was not stratified according to hormone receptor status. Future studies including biomarker analysis of BC1 and BC2 can hopefully shed some light on this issue and, more importantly, on the effect of adjuvant treatment after BC1 on tumour biology of BC2.

Although early detection of distant metastases does not affect survival or quality of life [29,30], recent studies suggest that early detection of local recurrences or CBC does improve prognosis [19,31,32]. However, results are not unanimous [33-35]. Previous studies are retrospective, leading to problems with lead-time (earlier detection results in a longer follow-up until event, even if the disease progression is the same) and length-time bias (slower growing tumours will be more easily detected, since they are detectable over a longer time period). To avoid lead-time bias we have looked at the risk of metastasis, using logistic regression, instead of DDFS with regard to mode of detection. Length-time bias may still be a problem in our study, although previous studies have not shown this to be a major source of concern [32,36-38]. We found that significantly fewer patients diagnosed with CBC within follow-up examinations subsequently developed metastases, which is probably to a large extent due to the fact that the tumours were discovered at an earlier stage. However, mode of detection remained a significant prognostic factor even after adjusting for tumour size, node status, and treatment for both tumours. This finding is in line with data from unilateral breast cancer, where diagnosis by screening mammography has been shown to be an independent prognostic factor even after adjustment for disease stage [39-42].

Presuming that surveillance after breast cancer diagnosis is effective, how long should it continue? In Sweden, a follow-up time of ten years was advocated until the mid-1990s. Today, clinical surveillance is often reduced to five years or less, followed by mammographic surveillance every 24 months within a screening program. In this study we found mode of detection to be associated with risk of metastasis even when BC2 was diagnosed more than ten years after BC1, suggesting that a long follow-up time could be of value.

The present study was based on a unique cohort, including over 700 patients with CBC, with information on patient and tumour characteristics, treatment, and outcome. Despite the large selection of patients and information included in this study, some potential sources of bias should be considered. For example, subgroup analysis will include fewer events. Inclusion in the cohort was based on data from the Swedish Cancer Register which is nationwide. However, some cases of CBC might still be missing or misclassified, and 150 charts were never found. Although data from the patients' individual charts is probably more reliable than register data, the quality of clinical notes and pathological records varied over time and between physicians. Due to the low annual incidence of contralateral breast cancer, a long period of follow-up was needed to receive a large cohort. Hence, standard surgical methods, routine histopathological analysis and adjuvant treatment have changed during the study period. The patients were included in the cohort based on diagnose date of the CBC. Hence, for women initially treated before 1977, there could be a selection of patients with a longer time interval to BC2. When adjusting our analyses for diagnose date of BC1 the multivariate analysis of time interval to BC2 including all patients and treatment was no longer significant. Otherwise significance in all other analysis remained constant.

## Conclusions

Patients with CBC are currently treated according to the tumour biology of BC1 and BC2 individually, and it is controversial whether prognosis after CBC is worse than after unilateral breast cancer [4,10-18]. Our study was based on clinical data from individual patients and results indicate that the time interval to BC2, especially for patients younger than 50 years, is a strong prognostic factor. Additionally, mode of detection was proven to be closely related to the risk of developing metastases. By taking time interval to BC2 and mode of detection into account when diagnosing patients with CBC, this study seems to have identified patients with a poor prognosis. Indeed, among patients diagnosed with symptomatic CBC within three years from BC1, more than 50% later developed metastases. Further translational studies of the tumour biology of BC2 in relation to time interval, adjuvant treatment after BC1 and mode of detection are needed to confirm and explain these results.

## Abbreviations

*BC1*: First breast cancer; *BC2*: Second breast cancer; *CBC*: Contralateral breast cancer; *CI*: Confidence interval; *DDFS*: Distant disease-free survival; *HR*: Hazard ratio; *IQR*: interquartile range.

## Competing interests

The authors declare that they have no competing interests.

## Authors' contributions

SA abstracted data from the individual charts, participated in database construction, performed statistical analysis and drafted the manuscript, POB was responsible for database construction and statistical analysis and participated in the manuscript draft, JM participated in the statistical analysis, interpretation of the data and manuscript draft. MF participated in the design of the study, interpretation of the data and manuscript draft. LR participated in the design of the study, was responsible for data abstraction, interpretation of the data and manuscript draft. All authors read and approved the final manuscript.

## Pre-publication history

The pre-publication history for this paper can be accessed here:

http://www.biomedcentral.com/1471-2407/11/114/prepub
